# Comparative evaluation of caries prevalence among group of Egyptian adolescents using DMFS and ICDASII methods: a cross-sectional study

**DOI:** 10.1186/s12903-023-02743-3

**Published:** 2023-01-24

**Authors:** Mohamed. H. Zaazou, Dalia Y. Zaki, Ali Abdelnabi, Tamer M. Hamdy, Reham S. Saleh, Shahinaz N. Hassan, Zeinab M. Zaki, Lamiaa M. Moharam

**Affiliations:** Restorative and Dental Materials Department, Oral and Dental Research Institute, National Research Centre (NRC), Dokki, Giza, 12622 Egypt

**Keywords:** Caries prevalence, ICDAS II, DMFS, Adolescents, Egypt

## Abstract

**Background:**

Limited data is available regarding the prevalence of dental caries as a chronic disease among adolescents using different caries assessment indices. The aim of this study was to compare and describe the prevalence of dental caries among group of Egyptian students using two caries assessment indices; DMFS and ICDAS II.

**Methods:**

This descriptive, cross-sectional epidemiological study included 2760 public secondary school students with age range from 15 to 18 years with permanent dentition and good general health. Presence of; retained teeth, congenital or developmental anomalies in the permanent dentition, orthodontic treatments, systematic conditions, smoking and general health problems were considered the exclusion criteria in this study. Participants were selected randomly from 8 public secondary schools in the Great Cairo, Egypt. The examination was achieved by **6** trained and previously calibrated examiners using sets of diagnostic mirrors, compressed air, a WHO probe and cotton rolls. DMFS index and ICDAS II system were used as caries detection methods. In DMFS index; the number of decayed (D), missing (M) and filled (F) surfaces was recorded, while in the ICDAS II index, the assessment of both cavitated and non-cavitated carious, missed and filled teeth with restorations /sealants was recorded. The examiners performed the oral examination using both scoring systems in an alternating manner. The collected data were explored for normality using Kolmogorov–Smirnov and Shapiro–Wilk tests. Chi square test was used to analyze the frequencies.

**Results:**

There was a statistical significant difference between the DMFS and ICDAS II methods results regarding the recorded number of caries affected teeth and cavitated teeth surfaces. The prevalence of dental caries among the investigated secondary school students was (**69.56%)** and (**78.29%)** for DMFS and ICDAS II, respectively.

**Conclusions:**

The prevalence of dental caries among Egyptian adolescent is high. ICDAS scoring system revealed higher caries prevalence values than DMFS method. ICDAS method is the best choice for the preventive goals, while DMFS is sufficient for clinical purposes.

## Background

Tooth decay is considered as the main public health problem all over the world. It is a multifactorial disease that is directly related to the interactions between behavioral, socioeconomic, genetic and microbiological factors [1]. Also, the international and macro-level play a crucial role in caries development through differences in the life style, the availability of health services and food choices [2]. That is why caries prevalence and severity are extremely variable worldwide and even in different region in the same country [3]. The prevalence of dental caries is much higher in the developing countries and steadily increases [3, 4].

Dental caries is considered as the most prevalent oral disease among school-aged students affecting about 60–90% of them globally [1]. Studies done on dental caries in adolescent is rising up, showing that the prevalence of dental caries is maximum at this critical age period [5]. This may be attributed to high sugar consumption, poor oral hygiene and general negligence of oral health at this age group. Moreover; dental caries is associated with tooth ache, or loss and school absenteeism, with subsequent negative impact on students' physical developmental and academic performance [6].

A wide variety of dental indices have been introduced to assess the prevalence of dental caries in a population. Selecting an effective method determines the quality of information obtained from epidemiological surveys [8].

The DMF method (DMFT(S)/dmfT(s) index) is the most known and most used method. It was presented by Klein and Palmer in 1938, as a method for detection of carious teeth among individuals and has been conducted for more than 90 years [8]. It is employed for both dentitions representing the total number of decayed (D), missing teeth due to caries (M) and filled teeth (F) or surfaces(S). Although this method has the benefit of being easily applied with a high level of validity and reliability, it is still unable to detect the pre-cavitation initial stages of the carious lesion within enamel [9, 10].

Lately, the International Caries Detection and Assessment System (ICDAS) in 2001, was allowed for researchers, clinicians, and epidemiologists to detect dental caries at different clinical stages [10]. This method was modified in 2005 into (ICDAS II) for caries assessment associated with restorations and sealants. In this system visual examination of teeth is done in a clean and completely dry environment for early carious lesions detection. ICDAS II is a two-digit coding technique; the first digit is made for sealants and restorations in teeth and has coding that ranges from (0 to 9). While, the second digit aimed to evaluate caries and detect early non-cavitated enamel lesions that appear after tooth surface dryness, with coding ranges from (0 to 6) [11–15].

Few studies about the prevalence of dental caries among the school students in Egypt are available and the determination of the exact burden of dental caries remains a considerable challenge. Thus, the aim of the current cross-sectional epidemiological study was to detect and compare the findings of dental caries prevalence among adolescents in the Great Cairo, Egypt using two different caries detecting systems; the DMFS and ICDAS II.

## Methods

### Study design and setting

This descriptive cross-sectional epidemiological clinical study was performed on eight public secondary school students with lower middle to lower socioeconomic class in the Great Cairo, Egypt. Great Cairo is the largest metropolitan area in Egypt, with a total population estimated at 20,901,000 [17]. The study was conducted over a period of two school years 2020–2022; from January 2020 to June 2022. The examination was planned to be fulfilled in one school year, but due to COVID 19 precautions schools were locked down for specific periods where the epidemic reached its peak.

### Ethical approval and parent informed consent

This study was carried out following the Declaration of Helsinki of the General Assembly in October 2013 (Seventh revision, 64th Meeting, Fortaleza, Ceará, Brazil) [16]. The study was approved by the Medical Research Ethical Committee (MREC) of National Research Centre (NRC); Cairo, Egypt (Reference number: 20157). The legal guardians of all participants were asked to sign a written informed consent form that was kept with the participant's records. Verbal informed consents were also taken from each participant.

### Eligibility criteria

Participants in this study were selected from eight Egyptians Public secondary school students to fulfill the following inclusion criteria; age range 15–18 years, students with permanent dentition, and good general health. While the exclusion criteria included; the presence of retained teeth, the existence of any congenital or developmental anomalies in the permanent dentition, orthodontic treatments, any systematic condition, smoking, general health problems, and participants with missed teeth due to any reason other than caries..

### Demographic data

The participants were all Egyptians (of one ethnic group), adolescents with an average age of 17.92 years. Both male and female and not married students were recruited for this study. The mean, standard deviation (SD) values and frequencies of demographic data was calculated.

### Sample size calculation

**Fig. 1:**
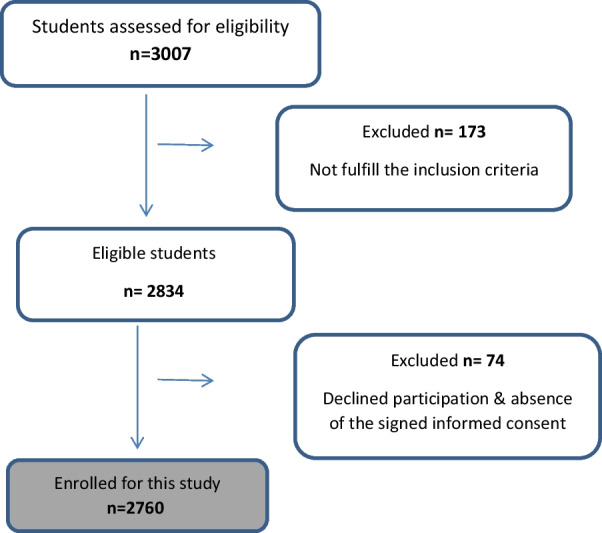
Flow chart showing participants selected for the cross-sectional study

Sample size calculation was done based up on previous similar cross-sectional study [17] according to the following sample size formula:

$${\text{n}} = {\text{ Z}}^{{2}} {\text{P}}\left( {{1} - {\text{P}}} \right)/{\text{d}}^{{2}} {\text{where}}:$$

where *n* is the sample size, *Z* is the statistic corresponding to the confidence level, P is expected prevalence, and d is the precision [18]. The minimum required sample size for this survey was calculated to be 2760 participants, with (95%) confidence interval and probability of statistical significance at 5%. However, a total of 3007 school students were invited for this survey. The number of the participating students was equally divided between the eight schools based upon the determined inclusion and exclusion criteria where 247 students were excluded from this study due to the absence of their legal guardians’ signed informed consents or they were not fulfilling the inclusion criteria (Fig. 1).

The sampling process was performed with a multistage cluster sampling (two-stage); where the students were divided into two clusters from higher to lower level culture at each stage. At the first stage, Great Cairo public secondary schools were divided into four main groups according to the school district (West, East, South and North). At the second stage, each main group was divided based on the gender into two subgroups (all-boys and all-girls schools). Furthermore, two schools were randomly selected from each district (one all-boys school and one all-girls school), that ends up with getting samples from eight public secondary schools in Great Cairo, Egypt.

### Examiners training and calibration

The oral examination was performed by six examiners using both scoring systems in an alternating manner; such that each examiner used one of the two investigated scoring systems for one week to be shifted to the other scoring system in the subsequent week. Training was done at National Research Centre (NRC), Cairo, Egypt in December 2019. For each examiner twelve hours of training were performed for each method, with a total of 24 h of examiners calibration. Guided by the ICDAS committee recommendations; the following training programs were applied to the examiners:

1. Power point presentations and discussions of the ICDAS II codes for examination.
2. Two days of examiners training included examination of a set of extracted teeth providing balanced numbers of tooth surfaces with ICDAS II codes. The examination findings of all examiners were reviewed to identify differences in interpretation. Examinations were repeated until agreement is reached among the examiners. All results were revised by a “senior examiner”.
3. Two days of reliability assessment using live subjects presenting with carious lesions with severity ranging between 1 to 6 (ICDAS). At least 20 patients were examined per each examiner and the “senior examiner”. Relationship and inter-examiner reliability between readings of different examiners was statistically analyzed.

### Participant's recruitment and clinical examination

A total of 2760 school students with age range 15–18 years were recruited at their school. The participants were selected from 8 different high schools in Great Cairo, Egypt. The examinations were conducted in the period from January 2021 to June 2022. Only students with their legal guardians’ signed informed consent were enrolled for the study.

The whole examination process was held in an operatory mobile dental bus with two fully equipped dental units in two separate compartments. All personal protective measures were followed strictly. Students were examined visually for dental caries by different examiners applying both ICDAS II and DMFS diagnosis systems for each student, so that each student has examination chart with the two forms. The teeth were first cleansed using brushes, rubber cups and prophylaxis paste to remove any food remnants and surface stains. Clinical examination was performed under standardized conditions using plain disposable dental mirrors, WHO probe and the dental unit light. For ICDAS II system, cotton rolls were used for moisture control followed by 5 s air dryness for all teeth using dental disposable plastic air–water syringe tips.

Each student was examined blindly by two examiners. The first examiner applied DMFS index, while the other examiner applied ICDAS II system for caries diagnosis. The examination of the teeth started with the right maxillary second molar moving anteriorly, passing through the left maxillary teeth, then the left mandibular teeth ending up with the right mandibular teeth.

### Dental caries detection methods

#### DMFS recording index

For the DMFS index, the examiners recorded a tooth as decayed only if a cavity had already been evident. A carious surface was verified once the WHO probe catches upon insertion with moderate and steady pressure, accompanied by presence of soft dentin at the base of the cavity and/or presence of an opacity adjacent to the examined area, indicating an undermined or demineralized enamel [22]. However, all caries stages that preceded the actual cavitation were considered sound. Regarding interproximal caries lesion detection method; the proximal surfaces of permanent teeth were examined using a blunt explorer, to avoid disrupting the enamel surface. However; Radio-visio-graphy (RVG) were used to confirm the diagnosis of interproximal caries when in doubt.

The numbers of the decayed (D), missed (M), and filled (F) surfaces were recorded in the DMFS form in the diagnostic charts. Carious lesion alone or both carious lesions and and restorations were recorded as “D”. Missing tooth due to caries was recorded as “M”. A permanent or temporary filling or tooth with only a defective filling but not decayed was counted as “F”. The third molar teeth were not considered. The DMFS index for recording is illustrated in Table 1.

**Table 1:** The DMFS index for recording

DMFS (permanent teeth)	
Decayed (D)	Cavitated carious lesion with exposed dentin either restoration present or not
Missing (M)	Tooth extracted due to caries
Filled (F)	Tooth with permanent or temporary restorations

#### ICDAS II recording system

Following the ICDAS guidelines, examinations were conducted with clean and dry tooth surfaces. Each affected surface took a two-digit code where the first digit represents the kind of restoration/ sealant of teeth and any other dental condition, while the second digit represent the caries level as reported in Table 2.

**Table 2:** Caries level of ICDAS II scoring system (second- digit code)

Criteria	Code
0	Sound tooth surface: no evidence of caries after prolonged air drying (5 s)
1	First visual change in enamel: opacity or discoloration (white or brown) is visible at the entrance to the pit or fissure after prolonged air drying, which is not or hardly seen on a wet surface
2	Distinct visual change in enamel: opacity or discoloration distinctly visible at the entrance to the pit and fissure when wet, lesion must still be visible when dry
3	Localized enamel breakdown due to caries with no visible dentine or underlying shadow: opacity or discoloration wider than the natural fissure/fossa when wet and after prolonged air drying
4	Underlying dark shadow from dentine + /– localized enamel breakdown
5	Distinct cavity with visible dentine: visual evidence of demineralization and dentine exposed
6	Extensive distinct cavity with visible dentine and more than half of the surface involved

The scoring criteria for the ICDAS II system were recorded as follows [24, 25]:

The restoration / sealant coding system of ICDAS II (first- digit code)

- 0 Surface not restored or sealed.
- 1 Sealant, partial.
- 2 Sealant, full.
- 3 Tooth colored restoration.
- 4 Amalgam restoration.
- 5 Stainless steel crowns.
- 6 Porcelain or gold or PFM crown or veneer.
- 7 Lost or broken restoration.
- 8Temporary restoration.
- 9 Used for the following conditions:
- 96 Tooth surface cannot be examined.
- 97 Tooth missing because of caries.
- 98 Tooth missing for reasons other than caries.
- 99 Unerupted.

### Statistical analysis

The collected data were loaded into a data entry program specially designed for epidemiologic surveys. Data were explored for normality using Kolmogorov–Smirnov and Shapiro–Wilk tests, data showed non-parametric (not-normal) distribution. Chi square test was used to analyze the frequencies. The significance level was set at *P* ≤ 0.05. Statistical analysis was performed with IBM® SPSS® Statistics Version 20 for Window.

## Results

Data from this survey included 2760 public school students, 45.36% (1252) were male and 54.64% (1508) were female with mean age 17.92 years. The participants were enrolled randomly in this study from eight public secondary schools in Great Cairo, Egypt, Table 3.

**Table 3:** The mean, standard deviation (SD) values and frequencies of demographic data

Variables	Demographic data		
	Mean/n	SD/%	*p*-value
Age	17.92	1.87	–
Gender			
Female	1508	(54.64%)	0.765 ns
Male	1252	(45.36%)	

### Inter-examiner reliability results

No-statistically significance difference was found between different readings, where (*p* = 0.550) with inter class correlation coefficient (ICC) higher than (0.900) in all readings, indicating a strong reliability and agreement between all examiner readings.

### Results for DMFs and ICDAS II scoring systems data

Data obtained after examination of the permanent dentition via DMFS and ICDAS II scoring systems is listed in Table 4.

**Table 4:** Results for DMFs data

Interpretation comparison	DMFs
Healthy sound teeth	68,640 88.81%
Filled teeth	400 (F)
Missed teeth	240 (M)
Non-cavitated teeth surface	
Teeth with initial enamel lesion after drying (first change)	Data cannot obtain
Teeth with enamel lesion with distinct change	Data cannot obtain
Cavitated teeth surface	
Localized enamel breakdown without visible change in dentin	8160 s (D)
Underlying dark shadow of dentine	
Slight cavity in dentine	
Extensive cavity in dentin	
Numbers of students	
Caries activity	1920
Free from caries activity	840
% of students	
Caries activity	69.56%
Free from caries activity	30.44%

### Comparison between DMFS and ICDAS II regarding the caries prevalence

The distribution of caries prevalence in the examined school students was recorded according to either presence or absence of caries lesion. Individual was the measurement unit of analysis (Table 5).

**Table 5:** The caries prevalence, total numbers and percentage of school students with or without caries lesions according to the DMFS and ICDAS II scoring system

	Students with caries lesion		Students without caries	
Scoring system	DMFS	ICDAS II	DMFS	ICDAS II
Numbers of students	1920	2161	840	599
% of students	69.56%	78.29%	30.44%	21.71%
*p*-value	< 0.001*			

^*^; significant (*p* < 0.005)

Results showed that the dental caries prevalence among the examined school students was **69.56%** and **78.29%** for DMFS & ICDAS II, respectively. Regarding the DMFS scoring system, 1920 (69.56%) students had teeth with carious lesions, meanwhile 840 (30.44%) students had caries-free teeth. Regarding the ICDAS II scoring system, 2161 (78.29%) students had carious teeth and 599 (21.71%) students had caries-free teeth. There was a statistically significant difference between both scoring systems where (*p* < 0.001) as shown in Table 6.

**Table 6:** The detailed relation and comparison of data obtained from DMFs and ICDAS II scoring systems

Interpretation comparison	DMFS	ICDAS II	*p*-value
Healthy (sound) teeth	68,640 88.81%	64,280 83.17%	0.504 ns
Filled teeth	400 (F)	400 (First digit 1 → 8)	1 ns
Missed teeth			
240 (M)	199 (due to caries 97) 41 (due to other 98)	1 ns	
Non-cavitated teeth surface			
Teeth surface with initial enamel lesion after drying (first change)	Data could not be obtained	601 s (second digit **1**)	
Teeth surface with enamel lesion with distinct change	Data could not be obtained	1720s (second digit **2**)	
cavitated teeth surface			
Localized breakdown of enamel without visible change in dentin	8188 s (D)	3976 s (second digit **3**)	0.723 ns
Underlying dark shadow of dentin		2842 s (second digit **4**)	
Slight cavity in dentin		958 s (second digit **5**)	
Extensive cavity in dentin		398 s (second digit **6**)	

### Comparison between DMFS and ICDAS II regarding classification of the tooth conditions:

The detailed relation between data obtained from DMFS and ICDAS II scoring systems regarding the means of sound teeth, filled teeth, missed teeth, decayed teeth, teeth with enamel lesion, localized enamel breakdown with or without visible dentin change, slight and extensive cavitated dentin are listed in Table 6. Regarding the employed DMFS Index and the ICDAS II system in the current study, teeth surfaces was the measurement unit of analysis.

Regarding the number and percentage of sound teeth recorded by the two methods; results showed that; the DMFS scoring system recorded 68,640 (88.81%) healthy sound teeth, while ICDASII scoring system recorded 64,280 (83.17%) healthy sound teeth, with no statistically significant difference between both scoring systems (*p* = 0.504). On the other hand; and regarding the number of cavitated teeth surfaces; the DMFS scoring system recorded higher number of cavitated teeth surfaces ( 8188 S) compared to ICDAS II scoring system which recorded a total cavitated teeth surfaces of (8174 s) (second digit- was recorded with score from 3 to 6). No statistically significant difference was found between both scoring systems (*p* = 0.723). However, the stage of carious lesions was only recorded in ICDAS scoring system.

There was no statistically significant difference between the two scoring systems regarding the data of missing and filled teeth. However, the cause of extraction, the type of restorative material and presence of sealant could only be recorded by ICDAS II scoring system (first- digit score). Moreover; for non- cavitated carious teeth surfaces; the ICDAS II system recorded a total of (2321 S) of non cavitated teeth surfaces (the second- digit score was 1&2), while DMFS index failed to record any early non- cavitated carious lesions.

### Comparison between DMFS and ICDAS II regarding the clinical application time:

Regarding the clinical application time, the ICDAS II system was more time consuming and required more clinical time, with a mean application time of 8.6 ± 2.4 min per student, while the DMFS index was less time consuming, with a mean application time of 4.5 ± 1.7 min per student.

## Discussion

This study was carried out in response to the deficiency in surveys data regarding the caries prevalence of adolescents in Egypt. Great Cairo area was chosen because it is the largest metropolitan area in Egypt, and is considered as the largest urban area in Africa and the Middle East [17]. Furthermore, Great Cairo has a substantial socio-economic discrepancy amongst its residents and districts. The socio-economic state of the students at Great Cairo area public secondary schools is expected to be beneath average or even low [18].

The study inclusion criteria targeted public secondary school students which represent a wide diversity of different standards of the community. Epidemiologic studies are considered an important method for specifying the requirements of any planned oral health program and preventive policies.

Dental caries detection is the most critical step in the treatment and prevention of such disease [19]. Epidemiologically, the most common technique for assessment of caries frequency is the DMFT(S)/dmft(s) index [4]. This method contains several limitations; as it provides only a numerical value that displays the teeth or surfaces which are decayed, missed or restored. No enough data about the caries state, stage, depth of penetration, restoration types and their conditions could be provided with this method. On the other hand, the simplicity and less-time consuming during the clinical application is a major advantage with this method. Moreover; comparability is considered another advantage; as DMF data can be compared with surveys from the 1940s onwards and compared to data from all over the world [20–23]. Nowadays, with advancement of minimal intervention principles, the possibility of treating carious lesions in early stages took a non-operative approach that avoided the complex and costly treatments. Consequently, the selection of the finest available caries assessment system that is able to record the non-cavitated lesions became compulsory.

The ICDAS system was introduced recently as a response to the shortcomings of the existing caries detection methods (DMF) with the capability to visually assess the patients’ tooth and restoration conditions more precisely[24, 26]. [19, 20].

Epidemiologic studies are considered an important method for specifying the requirements of any planned oral health program and different preventive policies. Therefore, this epidemiological cross-sectional study was conducted in an attempt to overcome the shortage in the data concerning caries prevalence among a sample of Egyptian adolescents at public secondary school students in Great Cairo area.

The results in this study showed that the caries prevalence among the examined students was 69.56% & 78.29% using DMFS & ICDAS II method, respectively. This reveals that the caries prevalence among the examined Egyptian adolescents is high as revealed by the two used caries detection methods. This might be attribute to the socio-economic level of the selected sample from public schools, that magnified the deficiency of proper oral hygiene knowledge's, the absence of appropriate preventive measures and the lack of regular dental examination. Moreover; high sugar consumption level and frequent eating of sticky food and carbohydrates may be considered as other contributing factors. Such findings are in line with other studies that estimated that caries prevalence in Egypt was about (60–70%)[20–23]. Also, this finding was in agreement with studies from Saudi Arabia, Iraq and India, which reported that caries prevalence among adolescents was 78.9%, 78.2% and 61.4%, respectively [4, 24, 25].

Moreover; in this study the recorded caries prevalence was statistically higher with ICDAS II method compared to DMFS method. This appears to be an expected outcome as ICDAS II include initial enamel carious changes among their criteria, which justifies why teeth are classified as sound by DMFS while classified of having enamel lesions by ICDAS system. In addition; the DMFS method seems to underestimate the presence of carious lesion and doesn’t represent the reality of the oral health condition, while the ICDAS II is able to provide more detailed and accurate information. This was in an agreement with Diniz et. al 2009 [20] who stated that the accuracy of the ICDAS system is comparable to the results obtained from Diagnodent device [20]. In this study the numbers of teeth recorded with initial enamel lesions (not cavitated) that couldn’t be recorded using DMFS system but only detected with ICDAS II system were 2321 teeth surfaces. This result was in agreement with Pitts 2008 who stated that DMF system does not provide information on the depth and the extent of caries] [14].

In this study the ICDAS II method was able to record the detailed condition of teeth, the extent of carious lesions and the state of restorations, clearly providing the specific part of care cycle which requires attention. while in the DMFS method the exact state and extension of the carious lesion wasn’t clear [26, 27]. Therefore, the proper line of treatment for such teeth could not be provided by the DMFS method. On the other hand, DMFS scores showed higher rates of cavitated surfaces compared to the ICDAS scoring. This might be attributed to the different type of examination performed by each test as the DMFS utilizes both visual and tactile senses in a visual-tactile examination procedure which seems to be more useful in detecting cavitated lesions [28]. Whereas the, ICDAS utilizes only visual examination under dry conditions which may be more efficient in detecting the first signs of enamel changes but didn’t seem to be as useful as the DMFS in detecting the cavitated lesions. In the same context, Qudeimat M.A et al. stated that the stage of tooth affection could determine the followed line of treatment [29]. Moreover, Bhoopath PH et al. proved that the ICDAS II system had a higher sensitivity for detecting non-cavitated carious lesions in comparison to DMF index [30].

In the same context, Qudeimat et. Al [29] stated that the stage of tooth affection could determine the followed line of treatment. Moreover, Bhoopathi et. al [30] have proved that the ICDAS II system had a higher level of sensitivity for detecting non-cavitated carious lesions in comparison to the DMF index.

The present study showed that the ICDAS II was more time consuming and require more clinical time for examination compared to DMFS. This is considers to be one of the limitations of the ICDAS II especially when used for epidemiological survey with a large number of populations. On the other hand, the time-saving application and simplicity make the DMF index the most used technique in large epidemiological surveys. This result was in agreement with Campus et al. [7] who found that DMFS was the fastest method for caries detection among all used methods; ICDAS, CAST and NYVADS Criteria [7].

The results of the present study come also in agreement with Melgar et al. 2016 [17]who compared the dmft/DMFT and ICDAS indices on children/adolescents and their mothers. Their results were consistent with the manifestation of a chronic disease, in children and adolescents. There were statistically significant differences because incipient or less severe lesions were more frequent and couldn’t be detected by the dmft/DMFT index, which lead to an underestimation of the disease in children and losing much information. Moreover; it was of great advantage to use ICDAS system with children because it presents a cost-effective method while in adults its use could be analyzed according to the available resources since its cost-effectiveness is lower. For this reason, it is important to consider the use of ICDAS in children and adolescents [20]. Limitation of the present study was in using only two caries detection methods (ICDAS II and the DMFS). Adding other methods as CAST and NYVADS criteria to this study could have added more to the main objective of the study. On the other hand, completion of this epidemiological study and examination of the targeted sample size under pandemic circumstances is quite a challenge and can be considered as one of the strengths of this study.

## Conclusions

Within the limitations of the present study, it could be concluded that; the prevalence of dental caries among Egyptian adolescents is high. ICDAS scoring system reveals higher caries prevalence values than DMFS method. ICDAS method is the best choice for preventive goals, while DMFS is sufficient for clinical goals.

### Recommendations

Increasing attention should be paid towards providing informative programs, preventive and therapeutic oral hygiene measures to the Egyptians adolescents. The term of the “best index” is considered to be a situation dependent. Conducting cross-sectional studies with larger sample size of populations and employing other caries detection methods would be beneficial. Longitudinal dental epidemiological research would be a greater investment in studying the prevalence of dental caries among different populations.

## Data Availability

The datasets generated during and/or analyzed during the current study are not publicly available due to institutional policy but are available from the corresponding author on reasonable request.

## Abbreviations

DMFS: Decayed, missing, and filled system
DMFT: Decayed, missing, and filled teeth
ICDAS: International caries detection and assessment system
MREC: Medical research ethical committee
NRC: National research centre
